# Factors Associated With Metabolic Syndrome in Korean Older Adults: A Cross‐Sectional Analysis of KNHANES VIII (2019–2021)

**DOI:** 10.1002/hsr2.72371

**Published:** 2026-04-19

**Authors:** Kumok Jang, GyeongAe Seomun

**Affiliations:** ^1^ College of Nursing, Korea University Seoul South Korea; ^2^ Transdisciplinary Major in Learning Health Systems Graduate School, Korea University Seoul Republic of Korea

**Keywords:** body mass index, energy intake, KNHANES, metabolic syndrome, older adults, smoking

## Abstract

**Background:**

Metabolic syndrome (MetS) is highly prevalent among older adults and substantially increases the risk of cardiovascular disease, type 2 diabetes, and mortality. Evidence regarding the independent contributions of lifestyle and nutritional factors to MetS in older populations remains inconclusive. This study examined factors associated with MetS among Korean older adults using nationally representative data within a complex‐sample analytic framework.

**Methods:**

This cross‐sectional study analyzed data from 3716 adults aged ≥ 65 years who participated in the Korea National Health and Nutrition Examination Survey (KNHANES) VIII (2019–2021). Survey weights, stratification, and clustering were incorporated to obtain nationally representative estimates. Complex‐sample multivariable logistic regression was used to estimate adjusted odds ratios (aORs) and 95% confidence intervals (CIs). Chronic disease variables overlapping with MetS diagnostic components were excluded from the primary model to minimize overadjustment. Sensitivity analyses and interaction models using continuous BMI (per 5 kg/m² increase) were conducted.

**Results:**

The weighted prevalence of MetS was 38.0%. In fully adjusted models, BMI demonstrated the strongest association with MetS. Compared with individuals with normal BMI, underweight participants had higher odds of MetS (aOR = 7.72, 95% CI: 3.23–18.49), whereas obese participants had lower odds (aOR = 0.27, 95% CI: 0.23–0.32). Female sex was associated with lower odds of MetS (aOR = 0.69, 95% CI: 0.53–0.90). Smoking was positively associated with MetS (aOR = 1.34, 95% CI: 1.03–1.74). Insufficient energy intake was associated with lower odds of MetS (aOR = 0.78, 95% CI: 0.65–0.95), whereas excessive intake was not statistically significant. Physical activity adherence, alcohol consumption, and dietary fiber intake were not independently associated. A significant interaction between continuous BMI and smoking status was observed (aOR = 1.47, 95% CI: 1.05–2.06).

**Conclusions:**

Among Korean older adults, BMI was the dominant factor associated with MetS within a nationally representative complex‐sample framework. The observed interaction between BMI and smoking suggests that adiposity and behavioral factors jointly influence metabolic risk in later life. These findings highlight the importance of integrated weight management and cardiometabolic risk reduction strategies in aging populations.

**Abbreviations**
aOR: adjusted odds ratio
BMI: body mass index
CI: confidence interval
KNHANES: Korea National Health and Nutrition Examination Survey
MetS: metabolic syndrome
OR: odds ratio

## 1 Introduction

South Korea has recently transitioned into a super‐aged society, defined by the United Nations as having ≥ 20% of the population aged ≥ 65 years [1]. According to national statistics from Statistics Korea, South Korea reached this threshold between late 2024 and early 2025, representing one of the fastest demographic transitions worldwide. Notably, the country required only approximately 7 years to progress from an aged society (≥ 14% aged ≥ 65 years) to a super‐aged society, an unprecedented pace among member countries of the Organization for Economic Co‐operation and Development [1].

This rapid population aging places substantial strain on public health systems, as it is accompanied by an increasing burden of age‐related chronic conditions and metabolic disorders. In particular, the growing prevalence of metabolic risk factors among older adults threatens healthy aging and healthcare system sustainability, highlighting the need for evidence‐based public health strategies tailored to Korea's rapidly aging population [1, 2].

Metabolic syndrome (MetS) is a major public health concern among older adults because it markedly increases the risk of cardiovascular diseases, type 2 diabetes, and all‐cause mortality [2, 3]. MetS is characterized by a cluster of metabolic abnormalities, including abdominal obesity, hypertension, hyperglycemia, and dyslipidemia, which collectively impair overall health and quality of life [4]. Given its increasing prevalence, identifying modifiable risk factors is essential for effective prevention and management [5].

Physical activity (PA) and dietary fiber intake are key lifestyle determinants of metabolic health. Regular PA improves glucose metabolism, cardiovascular function, and weight management [6, 7]. The World Health Organization (WHO) recommends at least 150 min of moderate‐intensity or 75 min of vigorous‐intensity aerobic PA per week for older adults [4, 8]. Although structured aerobic and resistance training can reduce cardiometabolic risk [9], adherence to PA guidelines remains low among older adults, and heterogeneity in type, intensity, and duration may influence metabolic outcomes [10, 11].

Dietary fiber intake is another important determinant of metabolic health. High‐fiber diets are associated with improved glycemic control, lipid metabolism, and weight regulation [12, 13]. Meta‐analyses suggest inverse associations between fiber intake and MetS risk; however, findings remain inconsistent in aging populations [14, 15]. Cultural dietary patterns and food composition may further modify these associations among Korean older adults [16]. In addition, fiber's metabolic effects may depend on fiber type, food matrix interactions, and gut microbiota composition [17, 18].

Although these factors have been extensively studied, their independent associations with MetS in older populations remain inconclusive when considered alongside anthropometric and health‐related factors [13, 15]. In later life, metabolic risk reflects complex interactions among lifestyle behaviors, nutritional status, body composition, and chronic disease burden. Sex‐based differences in metabolic regulation and health behaviors further suggest that associations may vary by sex, influenced by hormonal changes and age‐related physiological adaptations [14, 16, 17].

Body mass index (BMI), as a proxy for overall adiposity, is consistently identified as one of the strongest determinants of metabolic risk in later life [18, 19]. Beyond its direct association with MetS, BMI may modify relationships between behavioral factors and metabolic outcomes, underscoring the importance of evaluating these variables within an integrated analytic framework.

Methodologically, examining factors associated with MetS requires careful model specification. Chronic conditions such as hypertension, diabetes, and dyslipidemia overlap conceptually with MetS diagnostic components [4]. Statistical adjustment for these conditions may introduce overadjustment bias or conceptual redundancy, potentially obscuring independent associations. Analytic strategies must therefore account for confounding while avoiding circular adjustment.

Despite extensive research, few nationally representative studies have simultaneously examined BMI, lifestyle behaviors, and nutritional factors among Korean older adults using complex‐sample analytic approaches. Therefore, this study investigated factors associated with MetS among Korean older adults using data from the 8th Korea National Health and Nutrition Examination Survey (KNHANES VIII, 2019–2021). We evaluated BMI, lifestyle behaviors, and nutritional factors within a multivariable framework that incorporated complex sampling design and sensitivity analyses addressing conceptual overlap. By clarifying conditional associations within this framework, this study aims to inform targeted public health strategies to promote metabolic health in South Korea's rapidly aging population.

## 2 Methods

### 2.1 Study Design and Participants

This study used data from the 8th Korea National Health and Nutrition Examination Survey (KNHANES VIII, 2019–2021), a nationally representative cross‐sectional survey conducted by the [20] KNHANES employs a stratified, multistage probability sampling design, with enumeration districts as primary sampling units and households as secondary units.

A total of 22,559 individuals participated in KNHANES VIII (2019–2021). Participants aged < 65 years (*n* = 17,274) were excluded. Among the remaining 5285 adults aged ≥ 65 years, 182 individuals with missing data for any metabolic syndrome (MetS) component (waist circumference, blood pressure, triglycerides, fasting glucose, or HDL cholesterol) were excluded, yielding 5103 participants with complete MetS information.

**FIGURE 1:**
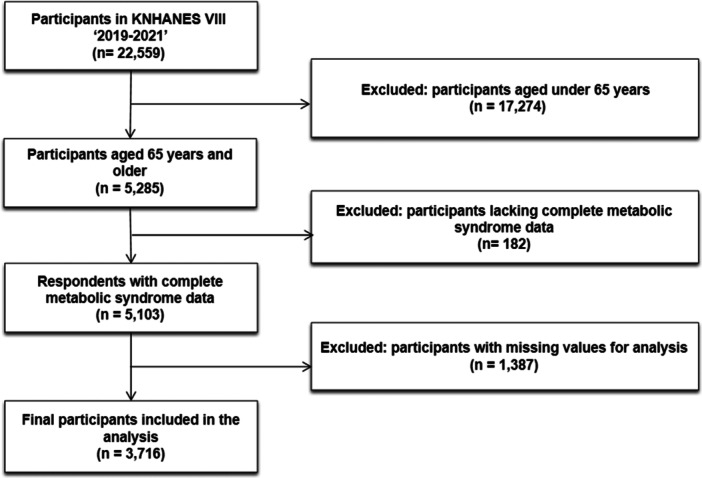
Flowchart depicting participant inclusion and exclusion criteria in the 8th Korea National Health and Nutrition Examination Survey (KNHANES VIII, 2019–2021).

An additional 1387 participants were excluded due to missing values in covariates included in multivariable models, resulting in a final analytic sample of 3716 older adults (Figure 1).

Missingness was primarily attributable to non‐participation in the nutrition survey component rather than incomplete anthropometric or laboratory measurements. Complete‐case analysis was applied to maintain consistency within the complex‐sample framework and preserve appropriate weighting and variance estimation.

To assess potential selection bias, age and sex distributions were compared between included and excluded participants; no material differences were observed.

### 2.2 Ethical Considerations

This study was conducted using publicly available, fully de‐identified KNHANES VIII data, ensuring participant anonymity and protection of personal information. As this study relied on secondary analysis of publicly available data, the requirement for informed consent was waived.

The study was conducted in accordance with the Declaration of Helsinki and reviewed and approved by the Institutional Review Board (IRB) of Korea University (approval number: KUIRB‐2024‐0046‐03). All procedures complied with relevant ethical standards.

### 2.3 Measurement of Variables

#### 2.3.1 Physical Activity

Physical activity was assessed using self‐reported frequency and duration of moderate‐ and vigorous‐intensity aerobic activities from the KNHANES VIII Health Interview Survey. Weekly minutes of physical activity were calculated by summing reported durations of moderate‐ and vigorous‐intensity activities across all relevant domains.

Physical activity adherence was defined according to the WHO recommendations. Participants were classified as adherent if they engaged in:

- ≥ 150 min of moderate‐intensity aerobic physical activity per week;
- ≥ 75 min of vigorous‐intensity aerobic physical activity per week; or
- An equivalent combination of moderate‐ and vigorous‐intensity activities.

Participants who did not meet these criteria were classified as non‐adherent.

#### 2.3.2 Dietary Fiber Intake

Dietary fiber intake was assessed using a single 24‐h dietary recall from the KNHANES VIII nutrition survey, which is conducted by trained interviewers. Nutrient intake was calculated using the Korean food composition database provided by the [20].

Dietary fiber intake was categorized according to the 2020 Dietary Reference Intakes for Koreans. Adequate fiber intake was defined as ≥ 25 g/d for men and ≥ 20 g/d for women. Participants were classified into:

- High fiber intake group: individuals meeting or exceeding the sex‐specific recommended intake;
- Low fiber intake group: individuals consuming less than the recommended intake.

#### 2.3.3 Metabolic Syndrome (Dependent Variable)

MetS was defined according to the National Cholesterol Education Program Adult Treatment Panel III (NCEP ATP III) criteria, with waist circumference cut‐off values adapted for the Korean population, consistent with the KNHANES application.

Participants were classified as having MetS if they met three or more of the following five criteria:

- Abdominal obesity: waist circumference ≥ 90 cm for men and ≥ 85 cm for women;
- Elevated blood pressure: systolic blood pressure ≥ 130 mmHg, diastolic blood pressure ≥ 85 mmHg, or current use of antihypertensive medication;
- Impaired fasting glucose: fasting plasma glucose ≥ 100 mg/dL or current use of antidiabetic medication or insulin;
- Hypertriglyceridemia: triglyceride levels ≥ 150 mg/dL or current use of lipid‐lowering medication;
- Low HDL cholesterol: < 40 mg/dL for men and < 50 mg/dL for women, or current use of lipid‐lowering medication.

Participants were categorized into two groups:

- Metabolic syndrome group: presence of ≥ 3 MetS components;
- Non‐metabolic syndrome group: presence of < 3 components.

#### 2.3.4 Covariates

Covariates were selected a priori based on prior literature and potential associations with metabolic syndrome (MetS).

Sociodemographic variables included sex, age group (65–74 years and ≥ 75 years), educational attainment (elementary school or less, middle school, high school, and college or higher), household income (low, lower‐middle, upper‐middle, and high), marital status (living with a spouse, separated, widowed, or divorced), and employment status (employed or unemployed).

Lifestyle‐related variables included smoking status (smoker or non‐smoker), alcohol consumption (drinker or non‐drinker), physical activity adherence (adherent or non‐adherent), and weight control experience during the past year (yes or no).

Nutritional variables included total energy intake, categorized as insufficient, adequate, or excessive based on estimated energy requirements, and dietary fiber intake (high vs. low) according to the 2020 Dietary Reference Intakes for Koreans.

Body mass index (BMI) was calculated as weight (kg) divided by height squared (m²) and categorized according to Asia‐Pacific criteria as underweight (< 18.5 kg/m²), normal weight (18.5–24.9 kg/m²), or obese (≥ 25.0 kg/m²). BMI was treated as the primary exposure variable in regression analyses.

Physician‐diagnosed hypertension, diabetes mellitus, and dyslipidemia were considered health‐related variables. Because these conditions overlap conceptually with diagnostic components of MetS, they were excluded from the primary multivariable model to minimize potential overadjustment. Their influence was evaluated in sensitivity analyses using alternative model specifications.

In additional models, BMI was also modeled as a continuous variable (centered and scaled per 5 kg/m² increase) to evaluate potential interaction effects.

All covariates were included to control for potential confounding rather than to imply causal relationships.

### 2.4 Statistical Analyses

All analyses were conducted using IBM SPSS Statistics version 27.0 (IBM Corp.) with complex‐sample procedures to account for the multistage stratified sampling design of KNHANES VIII. Sampling weights, stratification variables, and primary sampling units were incorporated to obtain nationally representative estimates and appropriate variance estimation.

Descriptive statistics are presented as unweighted frequencies and survey‐weighted percentages or weighted means with standard errors. Differences in MetS prevalence across covariate categories were evaluated using survey‐weighted chi‐square tests with Rao–Scott correction.

Multivariable associations were examined using complex‐sample logistic regression models to estimate odds ratios (ORs) and adjusted odds ratios (aORs) with 95% confidence intervals (CIs). The primary model simultaneously included sociodemographic, lifestyle, nutritional, BMI classification, weight control experience, and cancer diagnosis variables.

Multicollinearity among covariates was assessed using variance inflation factors (VIFs), and no evidence of problematic collinearity was identified.

Chronic disease variables (hypertension, diabetes mellitus, and dyslipidemia) were excluded from the primary model due to conceptual overlap with MetS diagnostic components. Sensitivity analyses were conducted by additionally including these variables to evaluate potential overadjustment.

To assess potential effect modification, an interaction term between continuous BMI (centered and scaled per 5 kg/m² increase) and smoking status was introduced in separate models. Statistical interaction was evaluated using Wald tests.

All regression analyses used a complete‐case approach; participants with missing values in variables included in the models were excluded. Additional sensitivity analyses were performed by (1) excluding underweight participants (BMI < 18.5 kg/m²) and (2) modeling total energy intake as a continuous variable.

All statistical tests were two‐sided, and *p* < 0.05 was considered statistically significant.

## 3 Results

### 3.1 General Characteristics and Health‐Related Factors of the Study Population

Table 1 summarizes the general characteristics and health‐related factors of the 3716 older adults included in the analysis. The weighted mean age was 72.55 ± 0.11 years. Overall, 63.4% (*n* = 2295) were aged 65–74 years, and 36.6% (*n* = 1421) were aged ≥ 75 years. Females comprised 52.8% (*n* = 2084) of the sample, while males accounted for 47.2% (*n* = 1632).

**TABLE 1:** General characteristics and health‐related factors of the study population (*n* = 3716).

Characteristics categories	*n* †	%‡(or mean ± SE)	Characteristics categories
Sociodemographic factors			
Sex	Male	1632	47.2
	Female	2084	52.8
Age (years)	mean ± SE	72.55 ± 0.11	
	65–74	2,295	63.4
	≥ 75	1421	36.6
Income Level	Low	1637	40.6
	Lower‐middle	1106	30.3
	Upper‐middle	613	18.0
	High	360	11.1
Education Level	≤ Elementary school	1973	49.9
	Middle school	644	18.0
	High school	710	20.7
	≥ College	389	11.4
Marital Status	Living with spouse	2542	70.5
	Separated	45	1.2
	Widowed	944	23.4
	Divorced	185	4.8
Employment Status	Unemployed	2282	61.9
	Employed	1434	38.1
Lifestyle factors			
Smoking Status	Non‐smoker	1397	40.4
	Smoker	2319	59.6
Alcohol Consumption	Non‐drinker	1959	50.9
	Drinker	1757	49.1
Physical Activity	Non‐adherent	2550	67.3
	Adherent	1166	32.7
Nutritional factors			
Energy Intake	Adequate	1818	49.0
	Insufficient	1463	38.9
	Excessive	435	12.1
Dietary Fiber Intake	High	2224	59.6
	Low	1492	40.4
Health‐related factors			
BMI (kg/m²)	mean ± SE	24.16 ± 0.07	
BMI Classification	Normal (18.5–24.9)	2196	60.3
	Underweight (< 18.5)	100	2.5
	Obese (≥ 25.0)	1369	37.2
Weight Control Experience	No	1657	42.6
	Yes	2059	57.4
Chronic Disease Status	No	955	26.6
	Yes	2761	73.4
Cancer Diagnosis Status	No	3313	89.0
	Yes	403	11.0
Metabolic Syndrome	No	2308	62.0
	Yes	1408	38.0

*Note:* All estimates were calculated using complex‐sample weights accounting for stratification, clustering, and sampling weights. BMI, body mass index; SE, standard error.

^†^ Unweighted sample size.

^‡^ Weighted percentage or weighted mean ± standard error.

Regarding socioeconomic characteristics, 40.6% (*n* = 1637) were in the lowest income group, 30.3% (*n* = 1106) in the lower‐middle group, 18.0% (*n* = 613) in the upper‐middle group, and 11.1% (*n* = 360) in the highest income group. Nearly half of the participants (49.9%, *n* = 1973) had an elementary school education or less, whereas 11.4% (*n* = 389) had a college education or higher. Most participants were living with a spouse (70.5%, *n* = 2542), followed by widowed individuals (23.4%, *n* = 944). A majority were unemployed (61.9%, *n* = 2282), while 38.1% (*n* = 1434) were employed.

In terms of lifestyle factors, 59.6% (*n* = 2319) were classified as smokers and 40.4% (*n* = 1397) as non‐smokers. Approximately half reported alcohol consumption (49.1%, *n* = 1757), whereas 50.9% (*n* = 1959) were non‐drinkers. Only 32.7% (*n* = 1166) adhered to recommended physical activity guidelines, while 67.3% (*n* = 2550) were classified as non‐adherent.

Regarding nutritional factors, 49.0% (*n* = 1818) had adequate energy intake, 38.9% (*n* = 1463) had insufficient intake, and 12.1% (*n* = 435) reported excessive intake. A greater proportion reported high dietary fiber intake (59.6%, *n* = 2224) compared with low intake (40.4%, *n* = 1492).

The weighted mean BMI was 24.16 ± 0.07 kg/m². Based on BMI classification, 60.3% (*n* = 2196) had normal weight, 37.2% (*n* = 1369) were obese, and 2.5% (*n* = 100) were underweight. More than half of the participants (57.4%, *n* = 2059) reported weight control experience. A substantial proportion had at least one chronic disease (73.4%, *n* = 2761), and 11.0% (*n* = 403) reported a history of cancer diagnosis.

Overall, 38.0% (*n* = 1408) of participants met the criteria for metabolic syndrome.

### 3.2 Differences in Characteristics According to Metabolic Syndrome Status

Survey‐weighted differences in general and health‐related characteristics by MetS status are presented in Table 2.

**TABLE 2:** Comparison of general characteristics and health‐related factors based on metabolic syndrome status (*n* = 3716).

Characteristics	Categories	Metabolic syndrome		Statistic† (Rao–Scott adjusted *F*)	*p*‐Value*
		No (*n* = 2308) *n* (%)	Yes (*n* = 1408) *n* (%)		
Sociodemographic factors					
Sex	Male	1079 (65.1)	553 (34.9)	9.525	*0.002*
	Female	1229 (59.3)	855 (40.7)		
Age (years)	65–74	1441 (62.7)	854 (37.3)	*1.026*	*0.312*
	≥ 75 years	867 (60.8)	554 (39.2)		
Income Level	Low	989 (60.4)	648 (39.6)	1.118	*0.341*
	Lower‐middle	694 (62.0)	412 (38.0)		
	Upper‐middle	393 (64.1)	220 (35.9)		
	High	232 (64.9)	128 (35.1)		
Education Level	≤ Elementary school	1156 (58.5)	817 (41.5)	*5.353*	*0.001*
	Middle school	408 (63.2)	236 (36.8)		
	High school	487 (67.3)	223 (32.7)		
	≥ College	257 (65.8)	132 (34.2)		
Marital Status	Living with spouse	1623 (63.4)	919 (36.6)	2.327	*0.073*
	Separated	29 (63.2)	16 (36.8)		
	Widowed	540 (57.8)	404 (42.2)		
	Divorced	116 (62.9)	69 (37.1)		
Employment Status	Unemployed	1403 (61.3)	879 (38.7)	0.931	*0.335*
	Employed	905 (63.1)	529 (36.9)		
Lifestyle factors					
Smoking Status	Non‐smoker	901 (63.2)	496 (36.8)	*0.996*	*0.319*
	Smoker	1407 (61.2)	912 (38.8)		
Alcohol Consumption	Non‐drinker	1,239 (62.6)	720 (37.4)	0.384	*0.536*
	Drinker	1069 (61.4)	688 (38.6)		
Physical Activity	Non‐adherent	1558 (60.4)	992 (39.6)	6.787	*0.009*
	Adherent	750 (65.5)	416 (34.5)		
Nutritional factors					
Energy Intake	Adequate	1125 (61.9)	693 (38.1)	*4.420*	*0.012*
	Insufficient	892 (60.1)	571 (39.9)		
	Excessive	291 (68.6)	144 (31.4)		
Dietary Fiber Intake	High	1387 (62.2)	837 (37.8)	*0.037*	*0.847*
	Low	921 (61.8)	571 (38.2)		
Health‐related factors					
BMI Classification	Normal (18.5–24.9)	1595 (72.6)	601 (27.4)	175.266	*< 0.001*
	Underweight (< 18.5)	94 (94.8)	6 (5.2)		
	Obese (≥ 25.0)	586 (42.6)	783 (57.4)		
Weight Control Experience	No	1021 (62.0)	636 (38.0)	*0.000*	*0.994*
	Yes	1287 (62.0)	772 (38.0)		
Chronic Disease Status	No	700 (72.9)	255 (27.1)	53.892	*< 0.001*
	Yes	1608 (58.1)	1153 (41.9)		
Cancer Diagnosis Status	No	2027 (61.0)	1286 (39.0)	*10.444*	*0.001*
	Yes	281 (70.5)	122 (29.5)		

*Note:* Percentages are weighted row percentages; frequencies (*n*) are unweighted.

* *p* values were derived from the Rao–Scott adjusted chi‐square test using the adjusted F statistic.

^†^ Rao–Scott adjusted F statistics accounting for stratification, clustering, and sampling weights.

#### 3.2.1 Sociodemographic Factors

MetS prevalence differed significantly by sex (Rao–Scott adjusted *F* = 9.525, *p* = 0.002), with higher prevalence among women (40.7%) than men (34.9%). Educational attainment was also associated with MetS (*F* = 5.353, *p* = 0.001), with the highest prevalence among participants with elementary school education or less (41.5%). Age group (*p* = 0.312), income level (*p* = 0.341), marital status (*p* = 0.073), and employment status (*p* = 0.335) were not significantly associated with MetS in unadjusted analyses.

#### 3.2.2 Lifestyle Factors

Physical activity adherence was significantly associated with MetS (*F* = 6.787, *p* = 0.009); prevalence was higher among non‐adherent participants (39.6%) than adherent participants (34.5%). Smoking status was not significantly associated in crude comparisons (38.8% among smokers vs. 36.8% among non‐smokers; *p* = 0.319), nor was alcohol consumption (38.6% among drinkers vs. 37.4% among non‐drinkers; *p* = 0.536).

#### 3.2.3 Nutritional Factors

Energy intake differed significantly by MetS status (*F* = 4.420, *p* = 0.012). Participants with excessive energy intake showed lower crude prevalence (31.4%) than those with adequate (38.1%) or insufficient intake (39.9%). Dietary fiber intake was not significantly associated (37.8% vs. 38.2%; *p* = 0.847).

#### 3.2.4 Health‐Related Factors

MetS prevalence differed markedly across BMI categories (*F* = 175.266, *p* < 0.001). Crude prevalence was 57.4% among obese participants, 27.4% among those with normal BMI, and 5.2% among underweight participants. Weight control experience was not associated with MetS in unadjusted analyses (*p* = 0.994).

Chronic disease status was strongly associated with MetS (*F* = 53.892, *p* < 0.001); prevalence was 41.9% among participants with chronic disease and 27.1% among those without. Cancer diagnosis status was also associated (*F* = 10.444, *p* = 0.001), with lower crude prevalence among those with a cancer history (29.5%) than those without (39.0%).

Given the small proportion of underweight participants (2.5%) and the limited number of MetS cases within this category (*n* = 6), crude survey‐weighted prevalence estimates for underweight participants should be interpreted cautiously. These unadjusted distributions describe population patterns and do not account for differences in demographic and behavioral composition across BMI categories; adjusted conditional associations are presented below.

### 3.3 Factors Associated with Metabolic Syndrome

Table 3 presents the results of complex‐sample multivariable logistic regression analyses examining factors associated with metabolic syndrome (MetS) among older adults. All estimates accounted for the stratification, clustering, and sampling weights of KNHANES VIII.

**TABLE 3:** Factors associated with metabolic syndrome among older adults (*n* = 3716). (complex‐sample multivariable logistic regression analysis).

Characteristics	Categories	Adjusted OR (95% CI)	*p*‐Value
Sociodemographic factors			
Sex	Male (ref.)		
	Female	0.69 (0.53–0.90)	0.006
Lifestyle factors			
Smoking Status	Non‐smoker (ref.)		
	Smoker	1.34 (1.03–1.74)	0.029
Alcohol Consumption	Non‐drinker (ref.)		
	Drinker	0.87 (0.73–1.04)	0.128
Physical Activity	Non‐adherent (ref.)		
	Adherent	0.89 (0.75–1.07)	0.223
Nutritional factors			
Energy Intake	Adequate (ref.)		
	Insufficient	0.78 (0.65–0.95)	0.011
	Excessive	1.19 (0.91–1.55)	0.208
Dietary Fiber Intake	High (ref.)		
	Low	1.15 (0.95–1.40)	0.149
Anthropometric factors			
BMI Classification	Normal (ref.)		
	Underweight (< 18.5 kg/m²)	7.72 (3.23–18.49)	< 0.001
	Obese (≥ 25.0 kg/m²)	0.27 (0.23–0.32)	< 0.001
Weight Control Experience	No (ref.)		
	Yes	1.16 (0.98–1.37)	0.079
Cancer Diagnosis Status	No (ref.)		
	Yes	1.33 (1.00–1.75)	0.048

*Note:* Chronic disease variables (e.g., hypertension, diabetes, and dyslipidemia) were excluded from the final multivariable model to avoid potential overadjustment and conceptual overlap with metabolic syndrome components.

Abbreviations: CI, confidence interval; OR, odds ratio; Ref., reference category. Adjusted for sex, income level, education level, marital status, employment status, smoking status, alcohol consumption, physical activity, energy intake, dietary fiber intake, BMI classification, weight control experience, and cancer diagnosis.

The fully adjusted model included sociodemographic, lifestyle, nutritional, anthropometric, weight control experience, and cancer diagnosis variables. Chronic disease status (hypertension, diabetes, or dyslipidemia) was excluded from the primary model to avoid conceptual overlap and potential overadjustment, as these conditions correspond to diagnostic components of MetS.

Female sex was independently associated with lower odds of MetS compared with male sex (aOR = 0.69, 95% CI: 0.53–0.90; *p* = 0.006). Smoking was independently associated with higher odds of MetS (aOR = 1.34, 95% CI: 1.03–1.74; *p* = 0.029). Alcohol consumption (aOR = 0.87, 95% CI: 0.73–1.04; *p* = 0.128) and physical activity adherence (aOR = 0.89, 95% CI: 0.75–1.07; *p* = 0.223) were not independently associated.

Compared with adequate energy intake, insufficient energy intake was associated with lower odds of MetS (aOR = 0.78, 95% CI: 0.65–0.95; *p* = 0.011), whereas excessive energy intake was not statistically significant (aOR = 1.19, 95% CI: 0.91–1.55; *p* = 0.208). Low dietary fiber intake was not independently associated (aOR = 1.15, 95% CI: 0.95–1.40; *p* = 0.149).

BMI classification showed the largest magnitude of association in the multivariable model. Compared with normal BMI, underweight participants had higher adjusted odds (aOR = 7.72, 95% CI: 3.23–18.49; *p* < 0.001), whereas obese participants had lower adjusted odds (aOR = 0.27, 95% CI: 0.23–0.32; *p* < 0.001). Weight control experience was not independently associated (aOR = 1.16, 95% CI: 0.98–1.37; *p* = 0.079). Cancer diagnosis history was associated with higher odds of MetS (aOR = 1.33, 95% CI: 1.00–1.75; *p* = 0.048). Because crude survey‐weighted prevalence estimates and aORs quantify different statistical estimands (unadjusted distributions vs. covariate‐conditional associations), adjusted associations should not be directly inferred from crude prevalence patterns.

#### 3.3.1 Sensitivity and Interaction Analyses

In sensitivity analyses excluding underweight participants, associations were materially unchanged; obesity remained inversely associated with MetS (aOR = 0.27, 95% CI: 0.23–0.32; *p* < 0.001) (Supplementary Table S1), indicating that findings were not driven by the small underweight subgroup.

When chronic disease status was additionally included, overall patterns of association were materially similar to the primary model (Supplementary Table S2). Given the conceptual overlap between chronic disease variables and MetS components, the coefficient for chronic disease status should be interpreted cautiously.

A statistically significant interaction between continuous BMI (per 5 kg/m² increase) and smoking status was observed (aOR = 1.47, 95% CI: 1.05–2.06; *p* = 0.025) (Supplementary Table S3). In stratified complex‐sample models, BMI (per 5 kg/m² increase) was inversely associated with MetS among both non‐smokers (aOR = 0.22, 95% CI: 0.17–0.29) and smokers (aOR = 0.30, 95% CI: 0.25–0.37).

## 4 Discussion

This study examined factors independently associated with metabolic syndrome (MetS) among Korean older adults using nationally representative KNHANES VIII (2019–2021) data and complex‐sample analytic procedures. The weighted prevalence of MetS was 38.0%. In fully adjusted models, BMI classification showed the strongest association with MetS, and smoking and insufficient energy intake were also independently associated, whereas alcohol consumption, physical activity adherence, and dietary fiber intake were not.

### 4.1 BMI and Metabolic Syndrome

BMI classification demonstrated the most pronounced association with MetS in this nationally representative sample of Korean older adults. In unadjusted survey‐weighted distributions, the crude prevalence of MetS was highest among obese participants (57.4%), followed by those with normal BMI (27.4%), and lowest among underweight individuals (5.2%). In contrast, after multivariable adjustment for demographic, socioeconomic, behavioral, and nutritional factors, obesity was inversely associated with MetS (aOR = 0.27, 95% CI: 0.23–0.32), whereas underweight status was associated with substantially higher odds (aOR = 7.72, 95% CI: 3.23–18.49).

The apparent reversal between crude prevalence patterns and adjusted associations reflects differences in statistical estimands rather than analytic inconsistency. Crude prevalence represents unadjusted survey‐weighted distributions within BMI categories, whereas aORs estimate covariate‐conditional associations after accounting for differences in population composition across BMI strata. Because BMI groups differed in age distribution, sex composition, and behavioral characteristics, adjustment for these covariates altered the conditional association between BMI and MetS.

The inverse association observed for obesity should not be interpreted as evidence of a protective biological effect. Instead, this pattern may reflect epidemiologic processes often described in aging research as the “obesity paradox.” In older populations, individuals with higher BMI may represent a selectively healthier subgroup who have survived despite adiposity, whereas individuals with normal or low BMI may include those with underlying frailty, chronic disease burden, or subclinical metabolic dysfunction. Survival bias and selective attrition may therefore influence cross‐sectional associations observed in later life.

Interpretation of BMI in older adults is further complicated by age‐related changes in body composition. BMI does not distinguish between fat mass and lean mass and does not capture fat redistribution or sarcopenic obesity, both of which are prevalent in aging populations. Consequently, BMI categories may not fully reflect true cardiometabolic risk profiles in this age group. Reverse causation is also plausible, as individuals with metabolic abnormalities or chronic illness may experience unintentional weight loss or modify body weight following diagnosis.

The underweight group comprised a small proportion of the sample and included only six MetS cases, resulting in wide confidence intervals. However, sensitivity analyses excluding underweight participants yielded materially similar findings, with obesity remaining inversely associated with MetS. This suggests that the primary BMI–MetS association was not driven solely by the small underweight subgroup.

Taken together, these findings should be interpreted as covariate‐conditional statistical associations within a multivariable framework rather than as evidence of a protective effect of obesity. Longitudinal studies incorporating direct measures of body composition are needed to clarify temporality and disentangle potential biological mechanisms from survival, selection, and reverse causation effects. Multicollinearity diagnostics indicated that variance inflation factors were below commonly accepted thresholds for all covariates. The primary exposure variable (BMI classification) demonstrated minimal collinearity (VIF = 1.02), suggesting that the magnitude and direction of the observed association were unlikely to be explained by model instability.

### 4.2 Smoking and Effect Modification by BMI

Smoking was independently associated with higher odds of MetS (aOR = 1.34, 95% CI: 1.03–1.74). In addition, a statistically significant interaction between continuous BMI (per 5 kg/m² increase) and smoking status was observed (aOR for interaction = 1.47, 95% CI: 1.05–2.06), indicating effect modification. In stratified models, BMI remained inversely associated with MetS in both non‐smokers (aOR = 0.22, 95% CI: 0.17–0.29) and smokers (aOR = 0.30, 95% CI: 0.25–0.37). Given the cross‐sectional design, these associations should be interpreted cautiously, as residual confounding, survival bias, and reverse causation cannot be excluded.

### 4.3 Energy Intake

Insufficient energy intake was associated with lower odds of MetS (aOR = 0.78, 95% CI: 0.65–0.95), whereas excessive intake was not independently associated. In older adults, total energy intake may reflect nutritional adequacy, health‐related dietary modification, or functional reserve rather than habitual long‐term intake. Accordingly, this inverse association should be interpreted cautiously and does not imply a protective effect. Longitudinal studies with repeated dietary assessments are needed to clarify temporality.

### 4.4 Physical Activity and Dietary Fiber Intake

Neither physical activity adherence nor dietary fiber intake remained independently associated with MetS after multivariable adjustment. Measurement limitations (self‐reported physical activity and single 24‐h dietary recall) may have attenuated associations. It is also plausible that part of the association between these behaviors and metabolic risk operates through adiposity, such that adjustment for BMI reduces their direct association with MetS.

### 4.5 Model Specification and Chronic Disease Variables

Hypertension, diabetes, and dyslipidemia were excluded from the primary model because they conceptually overlap with MetS diagnostic components, and adjusting for them may introduce overadjustment and circular inference. As a robustness check, sensitivity models including chronic disease status yielded overall patterns that were materially unchanged with respect to primary associations, supporting the appropriateness of the chosen model specification.

### 4.6 Strengths and Limitations

Strengths include the use of nationally representative KNHANES data and appropriate complex‐sample analytic methods accounting for stratification, clustering, and sampling weights. Several limitations warrant consideration. First, the cross‐sectional design precludes causal inference and does not establish temporality. Second, lifestyle and dietary variables were self‐reported, and dietary intake was assessed using a single‐day recall, which may not represent usual intake. Third, complete‐case analysis may have introduced selection bias if missingness was not completely at random; however, this approach was used to maintain analytic consistency within the complex survey framework. Finally, residual confounding cannot be fully excluded.

### 4.7 Implications

In this nationally representative sample of Korean older adults, BMI classification was most strongly associated with MetS, and smoking and energy intake also showed independent associations. These findings highlight the importance of weight‐related factors in metabolic health in later life and underscore the need to consider behavioral context, including smoking status and nutritional patterns, when interpreting metabolic risk among older adults.

## 5 Conclusion

This study identified factors independently associated with metabolic syndrome (MetS) among Korean older adults using nationally representative KNHANES VIII (2019–2021) data and complex‐sample analytic procedures. After accounting for stratification, clustering, and sampling weights, BMI classification demonstrated the strongest association with MetS. In adjusted models, underweight status was associated with higher odds of MetS, whereas obesity was associated with lower odds relative to normal BMI (Table 3). Differences between crude prevalence patterns and adjusted associations reflect underlying compositional differences across BMI categories rather than analytic instability.

Smoking and insufficient energy intake were independently associated with MetS, and a significant interaction between BMI and smoking was observed (Supplementary Table S3).

These findings should be interpreted within the limitations of a cross‐sectional design, and causal inference cannot be established. Exclusion of hypertension, diabetes, and dyslipidemia from the primary model was methodologically justified to avoid conceptual overlap with MetS components; sensitivity analyses including chronic disease status yielded materially similar results (Supplementary Table S2).

Overall, these results underscore the central role of body weight in metabolic health among older adults and highlight the importance of integrated risk management strategies in aging populations. Longitudinal studies are warranted to clarify temporal relationships and potential reverse causation.

## Author Contributions

**Kumok Jang:** conceptualization, methodology, investigation, formal analysis, writing – original draft, visualization. **GyeongAe Seomun:** conceptualization, supervision, writing – review and editing, funding acquisition, resources, project administration.

## Funding

The authors have nothing to report.

## Ethics Statement

This study was conducted in accordance with the Declaration of Helsinki and was approved by the Institutional Review Board of Korea University (IRB approval number: KUIRB‐2024‐0046‐03). This study used publicly available, fully de‐identified secondary data from the Korea National Health and Nutrition Examination Survey (KNHANES VIII). Accordingly, the requirement for written informed consent was waived by the Institutional Review Board. Approval was granted for secondary analysis of publicly available data.

## Conflicts of Interest

The authors declare no conflicts of interest.

## Transparency Statement

The lead author, GyeongAe Seomun, affirms that this manuscript is an honest, accurate, and transparent account of the study being reported; that no important aspects of the study have been omitted; and that any discrepancies from the study as planned (and, if relevant, registered) have been explained.

## Supporting information

Supporting File 1

Supporting File 2

Supporting File 3

Supporting File 4

## Data Availability

Data analyzed in this study are publicly available from the Korea National Health and Nutrition Examination Survey (KNHANES) website (https://knhanes.kdca.go.kr/). Additional processed data supporting the findings of this study are available from the corresponding author upon request.
