# Supplementary material for: Factors Associated With Metabolic Syndrome in Korean Older Adults: A Cross‐Sectional Analysis of KNHANES VIII (2019–2021)
Source: Health Sci Rep. 2026 Apr 19;9(4):e72371. doi: 10.1002/hsr2.72371 (PMC13092217; doi:10.1002/hsr2.72371)
Supplement: Supplementary file 1 — Supporting File 1 [file HSR2-9-e72371-s003.docx]

**Table S1.** Sensitivity analysis of factors associated with metabolic syndrome after excluding underweight participants (BMI <18.5 kg/m²).

| **Characteristics** | **Categories** | | **Adjusted OR (95% CI)** | | **p-value** |
| --- | --- | --- | --- | --- | --- |
| **Sociodemographic factors** | |  |  |  |  |
| Sex | | Male (ref.) |  |  |  |
|  | | Female |  | 0.68 (0.53–0.88) | .004 |
| **Lifestyle factors** | |  |  |  |  |
| Smoking Status | | Non-smoker (ref.) |  |  |  |
|  | | Smoker |  | 1.36 (1.04–1.76) | .021 |
| Alcohol Consumption | | Non-drinker (ref.) |  |  |  |
|  | | Drinker |  | 0.88 (0.74–1.05) | .171 |
| Physical Activity | | Non-adherent (ref.) |  |  |  |
|  | | Adherent |  | 0.88 (0.74–1.05) | .174 |
| **Nutritional factors** | |  |  |  |  |
| Energy Intake | | Adequate (ref.) |  |  |  |
|  | | Insufficient |  | 0.81 (0.67–0.97) | .026 |
|  | | Excessive |  | 1.22 (0.93–1.60) | .134 |
| Dietary Fiber Intake | | High (ref.) |  |  |  |
|  | | Low |  | 1.15 (0.94–1.39) | .159 |
| **Anthropometric factors** | |  |  |  |  |
| BMI Classification | | Non-obese (BMI <25.0) (ref.) |  |  |  |
|  | | Obese (BMI ≥25.0) |  | 0.27 (0.23–0.32) | <.001 |
| Weight Control Experience | | No (ref.) |  |  |  |
|  | | Yes |  | 1.15 (0.97–1.35) | .100 |
| Cancer Diagnosis Status | | No (ref.) |  |  |  |
|  | | Yes |  | 1.32 (0.99–1.74) | .052 |

Abbreviations: OR, odds ratio; CI, confidence interval; BMI, body mass index.

All models accounted for stratification, clustering, and sampling weights of KNHANES VIII.

Models were adjusted for age (continuous), sex, income level, education level, marital status, employment status, smoking status, alcohol consumption, physical activity, energy intake, dietary fiber intake, weight control experience, and cancer diagnosis status.

Underweight participants (BMI <18.5 kg/m²) were excluded.
