# Supplementary material for: Factors Associated With Metabolic Syndrome in Korean Older Adults: A Cross‐Sectional Analysis of KNHANES VIII (2019–2021)
Source: Health Sci Rep. 2026 Apr 19;9(4):e72371. doi: 10.1002/hsr2.72371 (PMC13092217; doi:10.1002/hsr2.72371)
Supplement: Supplementary file 2 — Supporting File 2 [file HSR2-9-e72371-s004.docx]

**TABLE S2.** Sensitivity analysis including chronic disease status in the multivariable complex-sample logistic regression model for metabolic syndrome (n = 3,716).

| **Characteristics** | **Categories** | | **Adjusted OR (95% CI)** | | **p-value** |
| --- | --- | --- | --- | --- | --- |
| **Sociodemographic factors** | |  |  |  |  |
| Sex | | Male (ref.) |  |  |  |
|  | | Female |  | 0.69 (0.53–0.90) | .006 |
| **Lifestyle factors** | |  |  |  |  |
| Smoking Status | | Non-smoker (ref.) |  |  |  |
|  | | Smoker |  | 1.33 (1.03–1.72) | .029 |
| Alcohol Consumption | | Non-drinker (ref.) |  |  |  |
|  | | Drinker |  | 0.86 (0.72–1.03) | .114 |
| Physical Activity | | Non-adherent (ref.) |  |  |  |
|  | | Adherent |  | 0.89 (0.74–1.07) | .229 |
| **Nutritional factors** | |  |  |  |  |
| Energy Intake | | Adequate (ref.) |  |  |  |
|  | | Insufficient |  | 0.79 (0.66–0.95) | .016 |
|  | | Excessive |  | 1.17 (0.89–1.53) | .256 |
| Dietary Fiber Intake | | High (ref.) |  |  |  |
|  | | Low |  | 1.15 (0.94–1.40) | .154 |
| **Health-related factors** | |  |  |  |  |
| BMI Classification | | Normal (ref.) |  |  |  |
|  | | Underweight (<18.5) |  | 7.08 (2.97–16.84) | <.001 |
|  | | Obese (≥25.0) |  | 0.28 (0.24–0.33) | <.001 |
| Weight Control Experience | | No (ref.) |  |  |  |
|  | | Yes |  | 1.16 (0.98–1.37) | .080 |
| Chronic Disease Status | | No (ref.) |  |  |  |
|  | | Yes |  | 0.64 (0.53–0.77) | <.001 |
| Cancer Diagnosis Status | | No (ref.) |  |  |  |
|  | | Yes |  | 1.29 (0.98–1.71) | .067 |

Note. OR, odds ratio; CI, confidence interval; Ref., reference category.

All analyses accounted for stratification, clustering, and sampling weights of KNHANES VIII.

The multivariable model was adjusted for age (continuous), sex, income level, education level, marital status, employment status, smoking status, alcohol consumption, physical activity, energy intake, dietary fiber intake, BMI classification, weight control experience, cancer diagnosis status, and chronic disease status (hypertension, diabetes, or dyslipidemia).

Chronic disease variables were included in this sensitivity model to evaluate potential overadjustment effects due to conceptual overlap with metabolic syndrome components. Results were materially unchanged compared with the primary model excluding chronic disease status.
