# Supplementary material for: Factors Associated With Metabolic Syndrome in Korean Older Adults: A Cross‐Sectional Analysis of KNHANES VIII (2019–2021)
Source: Health Sci Rep. 2026 Apr 19;9(4):e72371. doi: 10.1002/hsr2.72371 (PMC13092217; doi:10.1002/hsr2.72371)
Supplement: Supplementary file 3 — Supporting File 3 [file HSR2-9-e72371-s002.docx]

**Table S3.** Interaction between BMI (continuous) and smoking on metabolic syndrome (complex-sample logistic regression)

| **Variable** | **Adjusted OR (95% CI)** | | **p-value** |
| --- | --- | --- | --- |
| BMI (per 5 kg/m² increase) | 0.21(0.16-0.28) |  | *<.001* |
| BMI × Smoking | 1.47(1.05-2.06) |  | .025 |
| BMI among non-smokers | 0.22(0.17-0.29) |  | <.001 |
| BMI among smokers | 0.30 (0.25-0.37) |  | <.001 |

Footnotes

• BMI was modeled as a continuous variable scaled per 5 kg/m² increase (BMI5).

• Stratified odds ratios (BMI among non-smokers and BMI among smokers) were derived from separate complex-sample logistic regression models.

• All models were adjusted for age, sex, income level, education level, marital status, economic activity, alcohol consumption, physical activity, total energy intake, dietary fiber intake, weight control experience, and cancer diagnosis.

• All analyses accounted for stratification, clustering, and sampling weights.
